# New triazole-substituted triterpene derivatives exhibiting anti-RSV activity: synthesis, biological evaluation, and molecular modeling

**DOI:** 10.3762/bjoc.18.161

**Published:** 2022-11-09

**Authors:** Elenilson F da Silva, Krist Helen Antunes Fernandes, Denise Diedrich, Jessica Gotardi, Marcia Silvana Freire Franco, Carlos Henrique Tomich de Paula da Silva, Ana Paula Duarte de Souza, Simone Cristina Baggio Gnoatto

**Affiliations:** 1 Phytochemistry and Organic Synthesis Laboratory, School of Pharmacy, Federal University of Rio Grande do Sul, Porto Alegre, Brazil; 2 Clinical and Immunology Laboratory, Biomedical Research Institute, Pontifícia Universidade Católica do Rio Grande do Sul, Porto Alegre, Brazil; 3 Laboratory of Computational Pharmaceutical Chemistry, Faculty of Pharmaceutical Sciences of Ribeirão Preto, University of São Paulo, Ribeirão Preto, SP 14040-020, Brazil

**Keywords:** antiviral, betulinic acid, bioisosterism, respiratory syncytial virus, triterpene, ursolic acid

## Abstract

Respiratory syncytial virus (RSV) is a major cause of acute lower respiratory tract infections in infants. Currently, ribavirin, a nucleoside analog containing a 1,2,4-triazole-3-carboxamide moiety, is a first-line drug for its treatment, however, its clinical use has been limited due to its side effects. Here, we designed two new nitroaryl-1,2,3-triazole triterpene derivatives as novel anti-RSV drugs. Their anti-RSV and cytotoxic activity were evaluated in vitro, RSV protein F gene effects by RT-PCR and molecular modeling with inosine monophosphate dehydrogenase (IMPDH) were performed. Compound **8** was the best performing compound, with an EC_50_ value of 0.053 μM, a TI of 11160.37 and it inhibited hRSV protein F gene expression by approximately 65%. Molecular docking showed a top-ranked solution located in the same region occupied by crystallographic ligands in their complex with IMPDH. The results obtained in this study suggest that compound **8** might be a new anti-RSV candidate.

## Introduction

Respiratory syncytial virus (RSV), an enveloped RNA-type virus, usually causes cold-like symptoms and is considered the major cause of acute lower respiratory tract infections (ALRIs) in infants. Until 2019, RSV was a major cause of morbidity in the elderly and immunocompromised people. It has been linked to more than 40,000 hospitalizations and about 11,000 deaths in older adults every year in the United States [1]. It has been suggested that RSV was responsible for approximately 5.2% of under-five deaths globally [2–3]; however, during the SARS-CoV-2 pandemic, scientists and physicians have noticed that respiratory illness cases, like flu and RSV infections, were less compared to the previous years. In the United States, RSV season is usually between December and February; but in 2020–2021 there was no such peak. The same phenomenon could be observed in South Africa and Australia, where RSV season is also in winter; except that, in those countries, the 2020 peak came later than expected and, in the under-five range, there was an increase in reported cases, which caused a great deal of discussion about the causes of the anomaly. Some epidemiologists believe that this change to the numbers can be associated with COVID-19 precautions; however, they state that less RSV cases now could reduce immunity and they fear there will be a rebound in infections after the pandemic [4–7].

**Figure 1:**
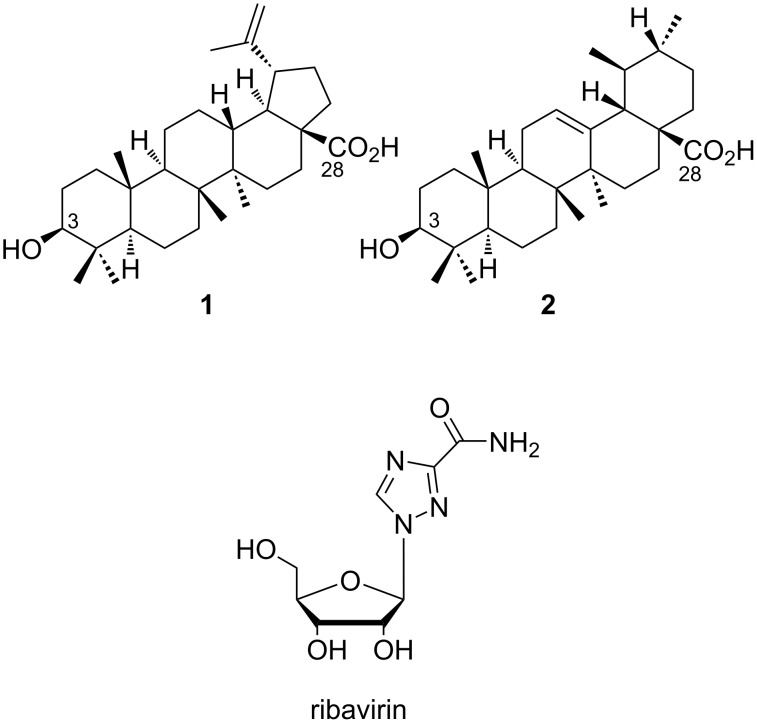
Structures of RBV, betulinic acid (**1**), and ursolic acid (**2**).

As a therapeutic resource, ribavirin, a nucleoside analog prodrug containing a 1,2,4-triazole-3-carboxamide moiety (RBV, Figure 1), is one of the few licensed drugs for treating RSV infections [8–9]. Although there are many suggested mechanisms of action, the main mechanisms for RBV involve the inhibition of the enzymes RNA-dependent RNA polymerase and inosine monophosphate dehydrogenase (IMPDH). IMPDH is required for the synthesis of guanosine triphosphate, which leads to a GTP depletion, thus, it prevents the replication of many RNA and DNA viruses [10]. Despite this, its efficacy has been controversial, as its use can cause hemolytic anemia, which is the leading cause for treatment being discontinued in 36% of real-life case studies [11]. Therefore, it is not indicated for patients with a history of or ongoing heart disease. In addition, RBV is also not indicated for patients with renal impairment, pregnant women, or those with autoimmune hepatitis [12–13].

In 1998, the FDA approved palivizumab for clinical use against RSV, a specific humanized monoclonal antibody that can reduce RSV-related hospitalizations, however, its use is only indicated for the prophylaxis of severe RSV infection in children. There have been discussions since early 2000 regarding the cost-effectiveness of this immunization and some researchers have suggested that adopting palivizumab should be restricted to only a few cases, such as patients with chronic lung disease or bronchopulmonary dysplasia, otherwise, the impact of the therapeutic agent is not significant [14–16].

Despite all these factors, the huge economic impact, the forecast of increasing numbers of RSV infections in the future, and the medical requirements associated with severe RSV infections, no vaccine or effective drugs have been developed so far, which reinforces the urgency for the research and development of a new type of anti-RSV drugs that can be widely adopted for clinical use [17].

Triterpenes are an extensive group of natural products, divided into several classes and presenting a huge variety of biological activities. For a long time, triterpenes have been receiving attention from the scientific community [18], especially for antiviral research, as glycyrrhizic acid, an oleanane, has demonstrated antiviral activity in the SARS-CoV-2 virus by inhibiting the ACE2 expression and SARS-CoV in vitro in Vero cells (IC_50_ = 365 μM), while chemical modifications to its backbone have been described which can enhance the potency of its antiviral activity [19–20]. Ursanes, represented by ursolic acid and lupanes, represented by betulinic acid, both natural pentacyclic triterpenes, are commonly found in several plant species could provide interesting prototypes for developing new antivirals, more specifically anti-RSV compounds [21]. Betulinic (**1**) and ursolic (**2**) acids (Figure 1) have demonstrated various antiviral activities, such as by HIV protease inhibition (IC_50_ = 8 and 9 μM) [22]. In addition, compound **1** has demonstrated some anti-SARS-CoV activity (EC_50_ = 10 μM; SI = >10) by having an inhibitory effect on the 3CL protease function [21,23]. Moreover, several studies have shown that semisynthetic triterpenes with modifications at the C-3 and C-28 positions might have the potential to be anti-human immunodeficiency virus type-1 (HIV-1) drugs [24–27]. Bevirimat, a betulinic acid derivative modified at the C-3 position, demonstrated secure, selective, and potent anti-HIV activity, however, it failed in phase IIb clinical trials due to viral resistance [28–31]. Even so, this class of natural products has great antiviral potential and its chemical modification could lead to new, efficient, and safe therapeutic resources.

In this scenario, here, we report our aims to develop new triterpene derivatives using a bioisosterism approach with RBV as an IMPDH inhibitor and anti-RSV agent.

## Results and Discussion

### Chemistry

In this study, we synthesized two new heterocycle-modified triterpene derivatives (compounds **7** and **8**) with a 1,2,3-triazole ring introduced by click chemistry in order to mimic the 1,2,4-triazole-3-carboxamide structure of RBV. This strategy was based on the bioisosteric relationship between both rings established in several papers [32–34]. Studies have made modifications at the C-3 and C-28 positions of triterpenes to synthesize 1,2,3-triazole derivatives via the Huisgen 1,3-cycloaddition reaction, but, as far as we know, this is the first report of the application of click chemistry to triterpenes with this objective [35–37].

Click chemistry is one of the most important tools used for the synthesis of biological compounds, including RBV derivatives [38–39]. Owing to the high yields, accessibility, and low cost, the click chemistry synthetic strategy is a promising option for use in medicinal chemistry studies [40].

**Scheme 1:**
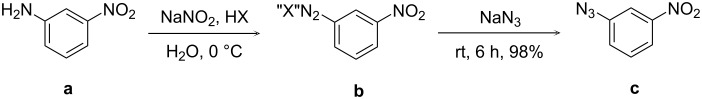
Synthesis of 1-azido-3-nitrobenzene (**c**).

The desired compounds **7** and **8** were obtained with yields of 68 and 59%, respectively. Initially, 1-azido-3-nitrobenzene (**c**) was obtained from *m*-nitroaniline (**a**) (Scheme 1) with excellent yields (98%), as previously described [41].

**Scheme 2:**
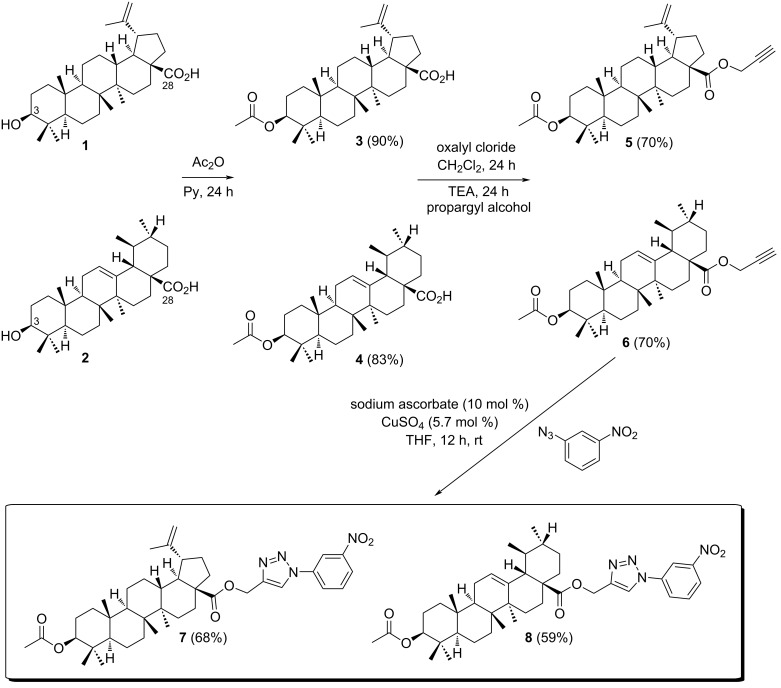
Synthesis of the triazole-substituted triterpene derivatives **7** and **8**.

The protection of the 3β-OH group of the triterpene skeleton was carried out by acetylation using acetic anhydride to prevent cleavage in acidic conditions, which resulted in the synthesis of derivatives **3** and **4**, with 90 and 83% yield, respectively (Scheme 2). The C-28-propargylated triterpene esters (**5** and **6**, 70% yield for both) of the acetate derivatives (**3** and **4**) were obtained. Finally, the reaction of derivatives **5** and **6** with meta-nitro-substituted azide (**c**) using click chemistry resulted in the synthesis of compounds **7** and **8** (Scheme 2).

### Biological assay

**Figure 2:**
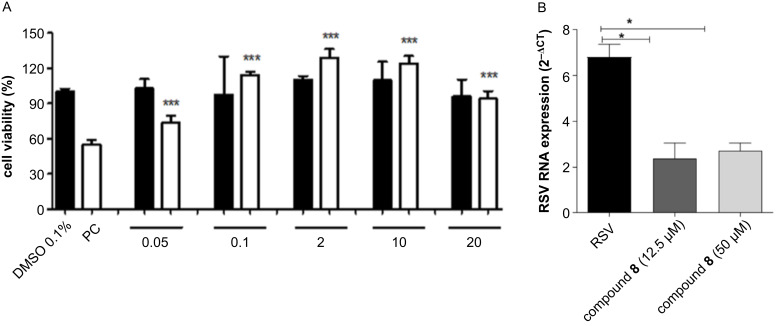
(A) Activity of compound **8** in A549 cells infected with RSV. MTT assay 96 h after treatment. DMSO (0.1%) was used as a negative control. A549 cells were infected with RSV and used as positive controls for infection (PC). Closed boxes represent non-infected A549 cells treated with compound **8**. Open boxes represent RSV-infected cells treated with compound **8**. (B) Viral load quantification by real-time PCR after treatment with compound **8**.

The anti-RSV activities of triterpene derivatives, intermediates, and scaffolds were evaluated using MTT and SRB assays in A549 cells treated 4 hours after viral infection (Table 1 and Figure 2). Both assays were compared and resulted in a statistically similar outcome, which showed a robust activity of the compounds tested. Ninety-six hours after infection, the antiviral activity was determined based on the RSV-induced death of the A549 cells and the viability of infected A549 cells after treatment, showing the anti-RSV or protective effect of the compounds. Also, we evaluated the cytotoxic effects of these compounds in VERO, HEP2, A549, and B16F10 cells. Control cells were treated with 1% dimethyl sulfoxide (DMSO), which was used to dilute the test compounds.

**Table 1:** Antiviral activity and cytotoxic effect of derivatives **1**–**8**.

	VERO	HEP2	B16F10	A549	A549+RSV				
compound	IC_50_ ± SD^a^	IC_50_ ± SD^a^	IC_50_ ± SD^a^	IC_50_ ± SD^a^	EC_50_ ± SD^b^	TI^c^	TI^d^	TI^e^	TI^f^

**1**	14.2 ± 0.2	28.0 ± 0.3	22.7 ± 0.8	17.8 ± 0.6	5.3 ± 0.7	3.4	2.7	5.3	4.3
**2**	12.9 ± 0.8	26.9 ± 0.4	16.2 ± 0.8	26.7 ± 0.9	17.3 ± 0.9	1.5	0.8	1.6	0.9
**3**	13.4 ± 2.1	11.5 ± 1.5	10.2 ± 1.1	53.0 ± 0.9	44.4 ± 0.5	1.2	0.3	0.3	0.2
**4**	12.6 ± 1.2	17.2 ± 0.9	12.4 ± 0.8	133.0 ± 1.1	14.3 ± 0.6	9.3	0.9	1.2	0.9
**5**	14.5 ± 0.2	18.4 ± 0.6	25.6 ± 0.5	88.8 ± 1	0.6 ± 0.8	148.0	24.2	30.7	42.7
**6**	26.1 ± 0.6	20.3 ± 0.4	29.0 ± 1.1	67.2 ± 0.5	36.2 ± 0.9	1.9	0.7	0.6	0.8
**7**	18.9 ± 1.4	23.8 ± 2	18.8 ± 0.7	42.7 ± 0.6	0.3 ± 0.1	142.3	63.0	79.3	62.7
**8**	21.6 ± 0.9	19.9 ± 1.2	9.8 ± 0.6	59.2 ± 0.9	0.05 ± 0.3	1184.0	432.0	398.0	196.0
RBV	nd	nd	nd	nd	4.9 ± 1.4	nd	nd	nd	nd

^a^Concentration (µM) that is toxic to 50% of non-infected VERO, HEP2, B16F10, and A549 cells by MTT test. ^b^Concentration (µM) that inhibits RSV replication by 50%. ^c^Therapeutic index (TI) = IC_50(A549)_/EC_50(A549 + RSV)_. ^d^Therapeutic index (TI) = IC_50(VERO)_/EC_50(A549 + RSV)_. ^e^Therapeutic index (TI) = IC_50(HEP2)_/EC_50(A549 + RSV)_. ^f^Therapeutic index (TI) = IC_50(B16F10)_/EC_50(A549 + RSV)_. nd = not determined.

Our results showed that the introduction of a 1-(3-nitrophenyl)-1,2,3-triazol-4-yl substituent in the C-28 position of both compounds **7** and **8** (EC_50_ = 0.314 and 0.053 µM, respectively) increased their antiviral activity, compared to that of the scaffolds **1** and **2** (EC_50_ = 5.3 and 17.3 µM, respectively) and acetylated compounds **3** and **4** (EC_50_= 44.4 and 14.29 µM, respectively). Although betulinic acid (**1**) exhibited greater antiviral activity than ursolic acid (**2**), the introduction of a nitroaryl-1,2,3-triazole substituent in **2** was more efficient than its introduction in scaffold **1**. Moreover, all tested compounds showed low cytotoxicity in VERO, HEP2, B16F10, and A549 non-infected cells. The therapeutic index (TI) is a comparison between the amount of a compound that causes a therapeutic effect and the amount that causes toxicity. Although all derivatives showed a reasonable cytotoxic effect in non-infected cells, compound **8** was the most efficient of them, with the highest TI of more than 1:1184 (results are summarized in Table 1). Our results are consistent with those of [42] for compounds **1** and **2** and show that our rational design was successful.

As result, the incorporation of a nitroaryl-1,2,3-triazole group into triterpenes resulted in more active and RSV-selective derivatives. The most active derivative (**8**) had a lower EC_50_ value against RSV than RBV (0.053 and 4.9 µM, respectively), which suggests that it might be a promising anti-RSV drug candidate. Compound **8** could control viral infection by preventing the proliferation of RSV in A549 cells, compared to the positive control (A549 cells infected with RSV without treatment). Furthermore, derivative **8** had low cytotoxicity in all non-infected cells tested, which is different to that observed for other derivatives where TI was expressively lower (Table 1 and Figure 2A).

The effect of compound **8** on RSV protein F gene expression was investigated using an RT-PCR assay. Total RNA was extracted from RSV-infected cells, both treated and untreated with compound **8** (12.5 and 50 µM). A real-time PCR was performed for the amplification of the RSV protein F gene using specific primers and probes: forward, 5'-AACAGATGTAAGCAGCTCCGTTATC-3'; reverse, 5'-GATTTTTATTGGATGCTGTACATTT-3'; and probe, 5'-FAM/TGCCATAGCATGACACAATGGCTCCT-TAMRA/-3', using human β-actin as an endogenous control gene using the TaqMan assay [43]. The delta cycle-threshold (ΔCt) was obtained by subtracting the endogenous control Ct value from the RSV protein F Ct value. Compound **8** reduced RSV protein F gene expression by approximately 65% at a concentration of 12.5 µM, compared to that of the control (Figure 2B). These results are in accordance with the EC_50_ value of compound **8**, verifying that compound **8** exhibited antiviral activity against RSV.

### Molecular docking

Therefore, owing to its excellent level of activity and lack of toxicity, evidenced by a high TI, we selected compound **8** for further studies, starting with the elucidation of the mechanism of action.

**Figure 3:**
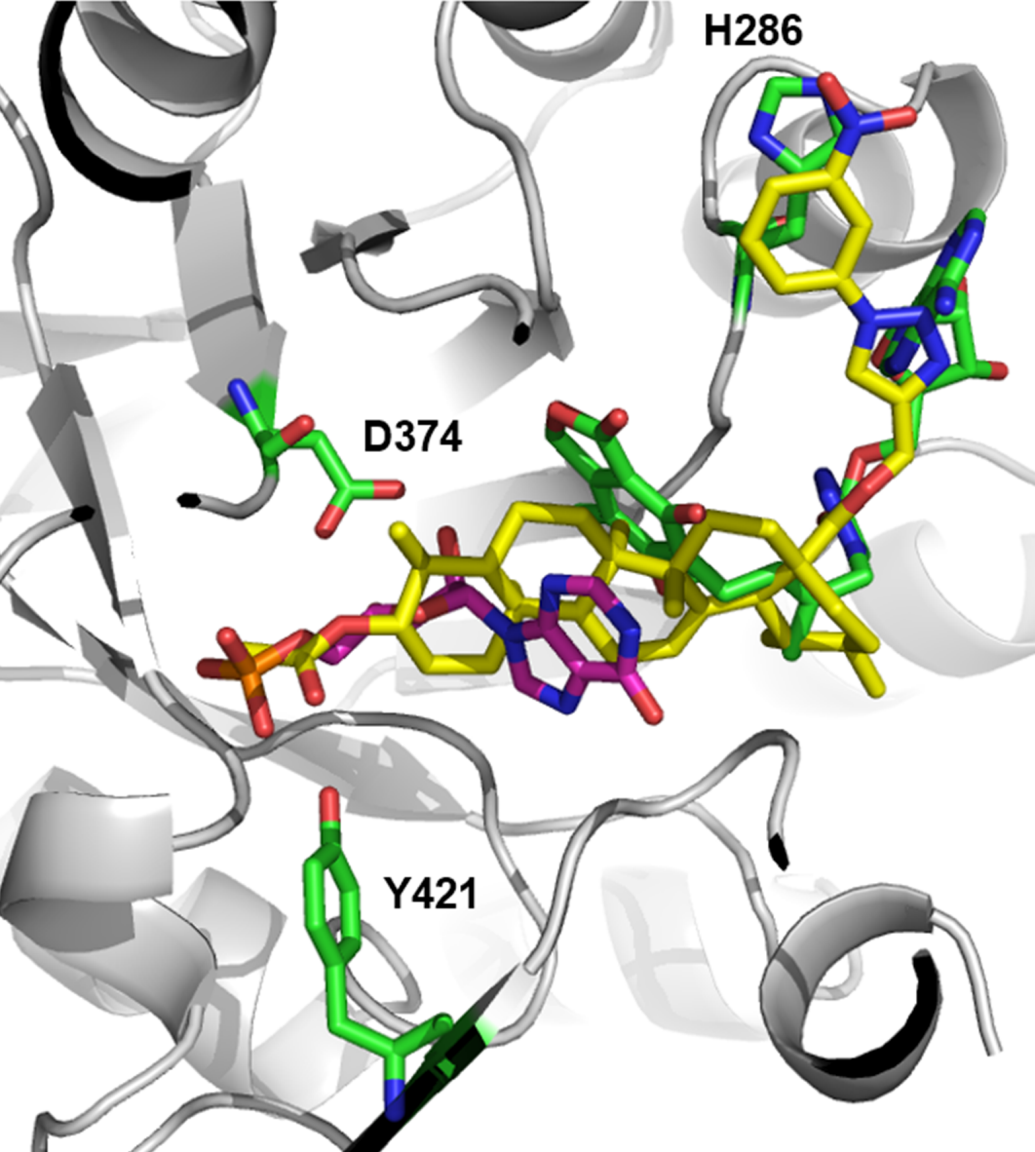
Superposition of the top-ranked docking solution of compound **8** (carbon atoms in yellow, in stick representation) with the crystallographic poses of IMP and inhibitor MAD1 (color by atoms in stick representation) inside the IMPDH active site from *Mycobacterium tuberculosis* (cartoon representation – PDB code 4ZQP), with selected residues and interactions here also represented.

Our hypothesis on the study of the mechanism of action relied on a comparison of compound **8** with crystallographic ligands of IMPDH, on the basis that it would represent a secure interpretation of the site interactions similarity with inhibitors, thus, suggesting that this compound acts by the same mechanism. Therein, flexible docking for compound **8** to the IMPDH protein from *Mycobacterium tuberculosis* (PDB code 4ZQP) was performed. After 10 docking runs, a top-ranked solution was identified and definitively located at the same region occupied by IMP and inhibitor MAD1, ligands of the crystallographic structure in complex with IMPDH. As can be seen in Figure 3, compound **8** (in yellow sticks) interacted through hydrogen bonds with Arg108 and Tyr421, represented by magenta dots, similar to the interactions observed in the crystallographic complex with IMP and the inhibitor MAD1, in cyan sticks. Moreover, the acetate group (at C-3 position) and nitroaryl-1,2,3-triazole (at C-28 position) of compound **8** mimicked the phosphate group and triazole ring of crystallographic ligands, respectively. In addition, the triterpene skeleton of compound **8** was located at the same region occupied by the aromatic rings of both IMP and inhibitor MAD1 ligands (Figure 3). One of the main known mechanisms of action of RBV is the depletion of intracellular GTP pools via the inhibition of cellular IMPDH induced by the 5-monophosphate metabolite of RBV [44].

Thus, our finding based on these results is that compound **8** may act similarly to IMPDH inhibitors and the active metabolite of RBV, leading to GTP depletion, since best poses achieved for the tested compound interact in an equivalent way as IMP and inhibitor MAD1 and in the same site of action, suggesting that compound **8** can be a bioisostere for IMPDH inhibitors that could be used to combat RSV infections. However, molecular modeling studies of the interaction of derivative **8** with human IMPDH can provide detailed information regarding the likely mechanism of antiviral action of this molecule.

## Conclusion

In this study, we synthesized new bioisosteric triterpene derivatives of RBV, containing the nitroaryl-1,2,3-triazole pharmacophore via click chemistry reaction catalyzed by copper. The introduction of a proper 1-(3-nitrophenyl)-1*H*-1,2,3-triazole substituent into triterpenes resulted in promising anti-RSV activity, compared to that of RBV, one of the few drugs available for treating RSV infections. Compound **8** was the most active in vitro derivative, even when compared to RBV. It could decrease the expression of RSV protein F at low concentrations and presented a low cytotoxic effect on non-infected cells compared to the same cell line infected with RSV. Furthermore, our previous results showed a strong interaction between the IMPDH protein and the derivative 8, indicating a probable mechanism of action. In summary, this study highlighted the importance of chemical modifications, in this case by linking a triazole ring to a triterpene backbone for the development of secure and efficient anti-RSV agents. Compound **8** could be a new anti-RSV drug candidate for preclinical studies. Furthermore, our results could represent important progress toward the development of new compounds to combat RSV, which particularly affects children, the elderly, and immunocompromised people. Despite being a preliminary outcome, the present study has advanced one more step in the development of new candidates and opens the door to new investigations about the real mechanism of action of triterpene derivatives against the RSV virus.

## Supporting Information

File 1 Experimental details of obtaining compounds **1** and **2** and experimental details for the preparation of compounds **3**–**8** and as well as the biological assays.
