# Supplementary material for: New triazole-substituted triterpene derivatives exhibiting anti-RSV activity: synthesis, biological evaluation, and molecular modeling
Source: Beilstein J Org Chem. 2022 Nov 9;18:1524–31. doi: 10.3762/bjoc.18.161 (PMC9663970; doi:10.3762/bjoc.18.161)
Supplement: File 1 — Experimental details of obtaining compounds 1 and 2 and experimental details for the preparation of compounds 3–8 and as well as the biological assays. [file Beilstein_J_Org_Chem-18-1524-s001.pdf]

BEILSTEIN JOURNAL OF ORGANIC CHEMISTRY

# Supporting Information

for

# New triazole-substituted triterpene derivatives exhibiting anti-RSV activity: synthesis, biological evaluation, and molecular modeling

Elenilson F. da Silva, Krist Helen Antunes Fernandes, Denise Diedrich, Jessica Gotardi, Marcia Silvana Freire Franco, Carlos Henrique Tomich de Paula da Silva, Ana Paula Duarte de Souza and Simone Cristina Baggio Gnoatto

*Beilstein J. Org. Chem.* **2022,** *18,* 1524–1531. doi:10.3762/bjoc.18.161

# Experimental details of obtaining compounds 1 and 2 and experimental details for the preparation of compounds 3–8 and as well as the biological assays

License and Terms: This is a supporting information file under the terms of the Creative Commons Attribution License (https://creativecommons.org/licenses/by/4.0). Please note that the reuse, redistribution and reproduction in particular requires that the author(s) and source are credited and that individual graphics may be subject to special legal provisions.
The license is subject to the *Beilstein Journal of Organic Chemistry* terms and conditions: (https://www.beilstein-journals.org/bjoc/terms)

## Chemistry

### General methods

All solvents were dried and distilled prior to use. Column chromatography (CC) was carried out on silica gel (Merck, Sao Paulo, Brazil, 60–230 mesh) using gradient eluent mixtures (cyclohexane/dichloromethane). $^{1}$H and $^{13}$C NMR spectra were recorded with a Varian Inova 500 NMR spectrometer. Chemical shifts are shown in parts per million (δ) with tetramethylsilane (TMS) as a reference. Data are reported as follows: chemical shifts, multiplicity (s = singlet, d = doublet, t = triplet, q = quartet, m = multiplet), coupling constants *J* (Hz), relative integration value. Mass spectra were recorded using an Ultrot of (Bruker Daltonics, SP, Brazil) mass spectrometer.

### Betulinic acid (1) and ursolic acid (2)

Betulinic acid (**1**) was obtained from the bark of *Platanus acerifolia L.* (maple) and its corresponding isomer ursolic acid (**2**) was obtained from *Malus domestica* peel (apple), as previously described [1].

### Obtention of synthetic compounds and characterization of the compounds 5–8

#### *1-Azido-3-nitrobenzene (c)*

This compound is obtained from 3-nitroaniline as a yellow crystalline powder in 98% yield. The reaction was conducted by a previously described methodology [2].

### 3-*O*-Acetylbetulinic acid (3) and 3-*O*-acetylursolic acid (4)

Compound **3** was prepared from **1** with 90% yield. Compound **4** was prepared from **2** with 83% yield as previously described [3-5].

#### *3-O-Acetyl-28-propargyl betulinic ester (5)*

To 3-*O*-acetylbetulinic acid (**3**, 0.25 g, 0.504 mmol) previously dissolved in 5 mL of dry dichloromethane, under inert atmosphere ($N_2$), oxalyl chloride was added (0.19 g, 1.5 mmol) under constant agitation for 24 h at room temperature to complete the acyl

chloride formation. After this, triethylamine (0.1515 g, 1.5 mmol) and propargylic alcohol (0.0841 g, 1.5 mmol) were added and kept under stirring for a further 24 h. Then, dichloromethane was removed under reduced pressure. Water (10 mL) was added to the crude product and extracted with dichloromethane (3 × 50 mL). The product was purified by column chromatography (cyclohexane/dichloromethane) yielding 70% of a white crystalline powder **5**. $^{1}$H NMR (500MHz, CDCl3): δ (ppm) 0.68 (1H, m, H- 5), 0.74 (3H, s, H-25), 0.81 (3H, s, H-24), 0.89 and 1.62 (1H each, m, H-1), 0.90 (3H, s, H-26), 0.96 (6H, s, H-27 and H-23), 1.12 and 2.43 (1H each, m, H-11), 1.21 and 1.99 (1H each, m, H-12), 1.25 and 1.90 (1H each, m, H-15), 1.36 and 1.54 (1H each, m, H-6), 1.37 and 2.18 (1H each, m, H-21), 1.38 (1H, m, H-9), 1.39 and 1.43 (1H each, m, H- 7), 2.20 (3H, s, H3CCOO), 1.44 and 2.22 (1H each, m, H-22), 1.48 and 2.22 (1H each, m, H-16), 1.62 (1H, m, H-18), 1.68 (3H, s, H-30), 1.78 (2H, m, H-2), 2.42 (1H, m, H-13), 2.43 (1H, s, -OCH2CCH), 2.48 (1H, m, H-19), 4.45 (1H, dd, H-3), 4.60 and 4.63 (1H each, s, H-29), 4.72 (2H, m, -OCH2-). $^{13}$C NMR (100 MHz, CDCl3): 14.55, 15.99, 16.18, 16.46, 18.16, 19.33, 20.85, 21.31, 23.68, 25.47, 27.92, 29.56, 30.48, 31.92, 34.24, 36.77, 37.09, 37.77, 38.29, 38.39, 40.80, 42.37, 46.85, 49.47, 50.46, 51.31, 55.41, 56.37, 74.31, 78.12, 80.91, 109.68, 150.37, 170.98, 175.15. HRMS m/z calcd. for [M+H] $C_{35}H_{52}O_4$ 537.3899 found 537.3934. As previously described [6].

**3-*O*-Acetyl-28-propargyl ursolic ester (6)**

This compound was prepared from 3-*O*-acetylursolic acid as described for **5**. It was obtained a white crystalline powder, with 70% yield.

$^{1}$H NMR (500MHz, CDCl3): δ (ppm) 0.83 (1H, s, H-25), 0.86 (1H, H-5), 0.92 (3H, s, H-24), 0.93 (3H, s, H-23), 0.94 (3H, d, H-30), 0.95 (1H, H-1), 1.05 (3H, d, H-29), 1.09 (3H, s, H-26), 1.12 (3H, s H-27), 1.32 (1H, H-19), 1.39 (4H, m, H-2 and H-26), 1.59

(6H, m, H-1, H-7, H-9, H-15, H-16 and H-21), 1.68 (1H, dt, H-6), 1.72 (1H, H-16), 1.91 (2H, H-22), 2.04 (1H, dt, H-15), 2.03 (3H, s, H3CCOO), 2.13 (1H, d, H-18), 2.51(1H, s, -OCH2CCH), 4.45 (1H, dd, H-3), 4.68 (2H, m, OCH2), 5.26 (1H, tl, H-12). $^{13}$CNMR (125 MZH CDCl3): 15.53, 16.72, 17.00, 17.17, 18.19, 21.14, 21.29, 23.29, 23.44, 23.59, 24.17, 27.98, 28.06, 30.60, 32.97, 36.41, 36.84, 37.66, 38.31, 38.9, 39.04, 39.41, 42.07, 47.47, 48.15, 51.55, 52.80, 55.28, 74.35, 78.10, 80.90, 125.74, 137.49, 170.98, 176.62. HRMS m/z calcd. for [M+H] $C_{35}H_{52}O_4$ 537.3899 found 537.3829.

**(1-(3-Nitrophenyl)-1*H*-1,2,3-triazol-4-yl)methyl 3-*O*-acetyl-betulinic acid ester (7)**

To 1-azido-3-nitrobenzene (**c**, 0.0157 g, 0.0957 mmol) dissolved in THF (5 mL) was added compound **5** (0.050 g, 0.0935 mmol) and distilled water (2.5 mL). After 10 min under constant stirring, it was added sodium ascorbate (1.95 mg, 10 mol %) and Cu($SO_4$) (0.89 mg, 5.7 mol %) in 2.5 mL of iced $H_2O$. The reaction was kept on stirring at room temperature for 24 h, then, extracted with water/dichloromethane (3 × 50 mL). The crude product was purified by column chromatography (cyclohexane/dichloromethane) obtaining a white crystalline powder 7, with 68% yield. $^1$H NMR (500 MHz, $CDCl_3$): δ (ppm) 0.69 (1H, m, H-5), 0.77 (3H, s, H-25), 0.81 (3H, s, H-24), 0.89 and 1.62 (1H each, m, H-1), 0.90 (3H, s, H-26), 0.96 (6H, s, H-27 and H-23), 1.18 and 2.46 (1H each, m, H-11), 1.21 and 1.99 (1H each, m, H-12), 1.25 and 1.90 (1H each, m, H-15), 1.36 and 1.54 (1H each, m, H-6), 1.37 and 2.18 (1H each, m, H-21), 1.38 (1H, m, H-9), 1.39 and 1.43 (1H each, m, H- 7), 2.22 (3H, s, H3CCOO), 1.44 and 2.22 (1H each, m, H-22), 1.48 and 2.22 (1H each, m, H- 16), 1.62 (1H, m, H-18), 1.68 (3H, s, H-30), 1.78 (2H, m, H-2), 2.42 (1H, m, H-13), 2.43 (1H, s, -$OCH_2CCH$-), 2.48 (1H, m, H-19), 4.45 (1H, dd, H-3), 4.59 and 4.71 (1H each, s, H-29), 7.26 (1H, s, NCH), 8.15 (1H, d, Ar-H), 8.20 (1H, s, Ar-H), 8.32 (1H, d, Ar-H),

8.58 (1H, t, Ar-H). $^{13}$C NMR (125 MHz, $CDCl_3$): 15.63, 15.96, 16.38, 18.02, 19.25, 20.81, 20.99, 21.25, 23.60, 25.40, 27.84, 29.65, 30.53, 31.90, 34.07, 36.80, 37.00, 38.31, 38.36, 40.64, 42.28, 46.97, 49.46, 50.32, 55.32, 56.79, 59.78, 80.82, 82.7, 109.75, 115.33, 121.43, 123.29, 125.93, 130.95, 137.58, 144.65, 148.95, 150.21, 171.08, 176.16. HRMS m/z calcd. for [M+H] $C_{41}H_{56}N_4O_6$ 701.4233 found 701.4239.

**(1-(3-Nitrophenyl)-1*H*-1,2,3-triazol-4-yl)methyl 3-*O*-acetyl-ursolic acid ester (8)**

Compound **8** was prepared as described for **7** using **6** as substrate. It was obtained a white powder with 59% yield.

$^{1}$H NMR (500 MHz, $CDCl_3$): δ (ppm) 0.85 (1H, s, H-25), 0.88 (1H, H-5), 0.92 (3H, s, H-24), 0.93 (3H, s, H-23), 0.94 (3H, d, H-30), 0.95 (1H, H-1), 1.05 (3H, d, H-29), 1.09 (3H, s, H-26), 1.12 (3H, s H-27), 1.32 (1H, H-19), 1.39 (4H, m, H-2 and H-26), 1.59 (6H, m, H-1, H-7, H-9, H-15, H-16 and H-21), 1.68 (1H, dt, H-6), 1.78 (1H, H-16), 1.96 ( 2H, H-22), 2.04 (1H, dt, H-15), 2.25 (3H, s, H3CCOO), 2.13 (1H, d, H-18), 4.45 (1H, dd, H-3), 5.28 (1H, tl, H-12), 7.25 (1H, s, NCH), 7.7 (1H, t, Ar-H), 8.1 (1H, d, Ar-H), 8.3 (1H, d, Ar-H), 8.58 (1H, s, Ar-H). $^{13}$C NMR (125 MHz, $CDCl_3$): 15.29, 16.65, 16.77, 16.99, 18.12, 21.13, 21.28, 23.20, 23.49, 24.13, 27.92, 28.02, 29.68, 30.57, 30.64, 32.84, 36.77, 37.61, 38.25, 38.79, 39.05, 39.51, 42.03, 47.39, 48.18, 52.87, 55.19, 80.84, 82.33, 109.91, 115.29, 122.52, 123.27, 125.63, 125.85, 139.95, 137.93, 144.53, 148.7, 170.89, 177.63. HRMS m/z calcd. for [M+H] $C_{41}H_{56}N_4O_6$ 701.4233 found 701.4287.

**Biological assays**

**Anti-RSV activity and cytotoxicity of compounds**

The anti-RSV activity was evaluated by infecting A549 cells with RSV virus followed by treatment with compounds **1–8**. Cell viability was determined by MTT and SRB assays by 96 hours of treatment. Dimethyl sulfoxide (DMSO) was used as vehicle control. We also assessed the cytotoxic effect of these compounds in A549, VERO, HEP2, and B16F10 cells not infected by the RSV virus, using the same methodology and treatment time used in the anti-RSV assay, in order to establish a selectivity parameter of the derivatives against the virus. Data from MTT and SRB experiments were expressed in $EC_{50}$ (µM) for the antiviral activity and $IC_{50}$ (µM) for the cytotoxic activity. The therapeutic index (TI) was calculated according to the previously described methodology by Visalli et al., and expressed as a ratio of the $IC_{50}$(VERO)/$EC_{50}$(A549 + RSV), $IC_{50}$(A549)/$EC_{50}$(A549 + RSV), $IC_{50}$(HEP2)/$EC_{50}$(A549 + RSV), $IC_{50}$(B16F10)/$EC_{50}$(A549 + RSV) [7].

**Cell culture**

A549 cells were kindly donated by Professor Dr. Fernando Spilki, Feevale, Brazil. Cells were cultured in DMEM (Cultilab) supplemented with 10% fetal bovine serum (FBS) (Cultilab), and 0.5 U/mL penicillin/streptomycin at 37 °C in a 5% $CO_2$ atmosphere at 100% humidity.

**Cultivation of RSV virus**

RSV A strain (line A2) was provided by Dr. Fernando Polack, Fundación Infant, Argentina. The virus was grown in A549 cells. Viral plaque-forming units (PFU) were identified using an RSV F protein-specific antibody (Millipore, Billerica, MA).

**Treatments**

Compounds **1**–**8** were dissolved in 1% of DMSO and culture medium. After reaching sub-confluence (70–80% of confluence), the cells were exposed to compounds at concentrations of 0.01 to 50 μM for antiviral activity and 5 to 100 μM for cytotoxicity for 96 h in DMEM 2% of FBS. Cells treated with DMSO (0.5% final concentration) were used as a negative control.

**Cytotoxicity assay**

**MTT assay**

The inhibition of cell proliferation by compounds was assessed using the MTT (3-(4,5-dimethylthiazol-2-yl)-2,5-diphenyltetrazolium bromide) assay. A549, VERO, B16F10 and HEP2 cells (5 × 103 cells/well in 190 μL medium per well) were seeded in a 96-well plate. After 24 h, the cells were treated with compounds **1**–**8** and incubated for 96 h. The optical density of each well was measured at 630 and 560 nm on an Envision microplate reader (PerkinElmer, Waltham, MA, USA). Four independent experiments were performed in triplicate for each test. The results were expressed as the percentage of cell viability where cells with no treatment were considered 100% viable. The concentrations of test compounds that resulted in 50% reduction of RSV-induced A549 cell death and the concentration that resulted in 50% cell death of A549 cell were determined by extrapolation, and the therapeutic indices (TI) were calculated.

**Sulforhodamine B assay for % growth inhibition**

The results obtained by MTT assay were confirmed using SRB (sulforhodamine B) assays. This assay is based on the SRB dye's ability to bind to cells previously basic protein attached to the culture plate with trichloroacetic acid (TCA). SRB is a dye with

two sulfonic groups that bind to basic amino acid residues under mildly acidic conditions and dissociate under basic conditions. A549, VERO, B16F10 and HEP2 cells (5 × 103 cells/well in 190 µL medium per well) were seeded in a 96-well plate. After 24 h, the cells were treated with compounds **1–8** and incubated for 96 h. The cells were fixed by adding 100 µL/well of 50% of TCA, for 60 min. The plates were washed five times in tap water and stained with 100 µL/well of SRB reagent (0.4% w/v SRB in 1% acetic acid) for 30 min. The plates were washed five times in 1% acetic acid to remove unbound SRB and allowed to dry overnight. SRB was solubilized with 100 µL of 10 mM Tris base/well of a 96 well plate with shaking for 5 min and the optical density of each well was measured at 630 and 560 nm on an Envision microplate reader (PerkinElmer, Waltham, MA, USA). Four independent experiments were performed in triplicate for each test. The results were determined by the plotting graph of the concentration vs. growth inhibition rate % and compared to MTT results.

**Viral load quantification by real-time PCR**

Real-time PCR analysis: A549 cells were seeded in a 24 well culture plate (5 × $10^4$ cells/mL) with DMEM low glucose containing 10% of FBS. After 24 h to complete adherence, cells were infected with RSV (104 PFU/mL) for 2 h and then treated with compound **8** at two different concentrations, 12.5 µM and 50 µM. The cells were kept in DMEM low glucose containing 5% of FBS for 96 h until gene expression analysis. Next, cells were harvested and total RNA was extracted using TRIzol reagent (Ambion™, Thermo Fisher Scientific, Waltham, MA, USA) following the manufacturer's instructions and complementary DNA was synthesized using the reverse transcriptase kit GoScrip™ (Promega™, Madison, WI, USA). The real-time PCR was carried out using 4 ng of samples cDNA template, using specific primers

and probes for TaqMan Assay (Applied Byosistems, Thermo Fisher Scientific, Waltham, MA, USA). For amplification of the RSV F protein gene, we used specific primers and probes: forward-5'-AACAGATGTAAGCAGCTCCGTTATC-3', reverse-5'-GATTTTTATTGGATGCTGTACATTT-3' and probe 5'-FAM/TGCCATAGCATGACACAATGGCTCCT-TAMRA/-3' and human β-actin (Hs00174103_m1 ACTB) as an endogenous control gene. PCR conditions were recommended by GoTaq™ Probe qPCR Master Mix protocol (Promega™, Madison, WI, USA). Quantification of gene expression was conducted using StepOne™ (real-time PCR system; Applied Biosystems). The $2^{(-\Delta\ CT)}$ method was used to analyze real-time PCR reaction data as previously described [8].

**Statistical analysis**

Data were presented as mean ± standard deviation of three individual experiments. Statistical analysis was performed by a one-way ANOVA analysis through the Prism statistical software package (GraphPad Software, Inc., La Jolla, CA, USA). $*p < 0.05$ was considered statistically significant.

**Docking procedures**

The crystal structure of inosine monophosphate dehydrogenase from *Mycobacterium tuberculosis* in complex with inosine 5'-monophosphate (IMP) and a third-generation mycophenolic adenine nucleotide inhibitor MAD1 (PDB code 4ZQP, at 1.9 Å resolution) [9] has been considered for flexible docking with compound **8**, and the calculations were carried out using the GOLD (Genetic Optimisation for Ligand Docking) 5.2.2 software [10]. GOLD was comprehensively validated, reliably identifying the correct binding mode for a large range of test set cases, in a vast set of independent studies, with a rate of success in 70–80% of the PDB protein–ligand structures thus analyzed, such as reported in the literature [11].

A parameters set including a population of 100 conformers, 100000 operations, 95 mutations and 95 crossovers has been here used. The simulations were then performed inside a selected region of the enzyme active site (sphere of 11 Å radius, centered at $x = -40.08$, $y = -10.52$, $z = 2.09$). The number of docking simulations to be performed with the ligand was specified under 10 GA (genetic algorithm) runs, once each docking run can evolve to different ligand poses (pose = conformation + orientation). Thus, ten poses of the highest score (top-ranked GOLD solutions) obtained for compound **8** were selected by using the CHEMPLP score function. In this case, a Piecewise Linear Potential (fPLP) is used to model the steric complementarity between protein and ligand, and for CHEMPLP the distance- and angle-dependent hydrogen and metal bonding terms from other fitness function also implemented in GOLD, so-called ChemScore, are considered. CHEMPLP has been found to give the highest success rates for docking pose prediction as well as virtual screening experiments against diverse validation test sets and it was here chosen as the fitness function. Based on this CHEMPLP function, GOLD classifies the orientation of the compound **8** by decreasing the order of affinity (the scores) with the binding site of the receptor.

Previous to the docking calculations and after the removal of the ligand as well as crystallographic water molecules from the complex structure here used, hydrogen atoms of the residues side chains were added and oriented in the active site region. Besides, suitable 3D structures and charges of the ligand compound **8** were previously built and optimized using molecular mechanics (MMFF force field), followed by the AM1 method, thus implemented at the Spartan'06 software.

**References**


1. Innocente, A. M.; Silva, G. N. S.; Cruz, L. N.; Moraes, M. S.; Nakabashi, M.; Sonnet, P.; Gosmann, G.; Garcia, C. R. S.; Gnoatto, S. C. B. *Mol. 2012, Vol. 17, Pages 12003-12014* **2012**, *17* (10), 12003–12014. https://doi.org/10.3390/MOLECULES171012003.
2. Zhang, F.; Moses, J. E. *Org. Lett.* **2009**, *11* (7), 1587–1590. https://doi.org/10.1021/OL9002338/SUPPL_FILE/OL9002338_SI_001.PDF.
3. Waechter, F.; da Silva, G. N. S.; Willig, J. B.; de Oliveira, C. B.; Vieira, B. D.; Trivella, D. B. B.; Zimmer, A. R.; Buffon, A.; Pilger, D. A.; Gnoatto, S. C. B.. *Agents Med. Chem.* **2017**, *17* (13). https://doi.org/10.2174/1871521409666170412143638.
4. Innocente, A.; Casanova, B. B.; Klein, F.; Lana, A. D.; Pereira, D.; Muniz, M. N.; Sonnet, P.; Gosmann, G.; Fuentefria, A. M.; Gnoatto, S. C. B.. *Chem. Biol. Drug Des.* **2014**, *83* (3), 344–349. https://doi.org/10.1111/cbdd.12251.
5. Sidova, V.; Zoufaly, P.; Pokorny, J.; Dzubak, P.; Hajduch, M.; Popa, I.; Urban, M.. *PLoS One* **2017**, *12* (2). https://doi.org/10.1371/JOURNAL.PONE.0171621.
6. Chao, W.; Lu, L.; Heya, N.; Xiangpeng, L.; Qian, W.; Xifeng, J.; Xiaoyu, X.; Fei, Y.; Tianhong, Z.; Jinglai, L.; Zhenqing, Z.; Baohua, Z.; Guodong, L.; Lifeng, C.; Shibo, J. and Keliang, L.; J. Med. Chem. 2014, 57(17), 7342-7354.
7. Visalli, R. J.; Ziobrowski, H.; Badri, K. R.; He, J. J.; Zhang, X.; Arumugam, S. R.; Zhao, H. *Bioorg. Med. Chem. Lett.* **2015**, *25* (16), 3168–3171. https://doi.org/10.1016/J.BMCL.2015.05.099.
8. Livak, K. J.; Schmittgen, T. D. *Methods* **2001**, *25* (4), 402–408. https://doi.org/10.1006/METH.2001.1262.
9. Makowska-Grzyska, M.; Kim, Y.; Gorla, S. K.; Wei, Y.; Mandapati, K.; Zhang,

M.; Maltseva, N.; Modi, G.; Boshoff, H. I.; Gu, M.; Aldrich, C.; Cuny, G. D.; Hedstrom, L.; Joachimiak, A. *PLoS One* **2015**, *10* (10), e0138976. https://doi.org/10.1371/JOURNAL.PONE.0138976.

10. Verdonk, M. L.; Cole, J. C.; Hartshorn, M. J.; Murray, C. W.; Taylor, R. D. *Proteins Struct. Funct. Bioinforma.* **2003**, *52* (4), 609–623. https://doi.org/10.1002/PROT.10465.

11. Hartshorn, M. J.; Verdonk, M. L.; Chessari, G.; Brewerton, S. C.; Mooij, W. T. M.; Mortenson, P. N.; Murray, C. W. *J. Med. Chem.* **2007**, *50* (4), 726–741. https://doi.org/10.1021/JM061277Y.
